# Transvenous lead extractions in a single high-volume center over a 24-year period: High success rate and low complication rate

**DOI:** 10.1016/j.hroo.2023.01.003

**Published:** 2023-01-20

**Authors:** Thomas Morgan Knutsen, Per Torger Skretteberg, Paul Vanberg, Ziaullah Kamal, Sigrun Halvorsen, Knut Liestøl, Torkel Steen, Eivind Platou

**Affiliations:** ∗ Department of Cardiology, Oslo University Hospital, Ullevaal, Oslo, Norway; † Institute of Clinical Medicine, University of Oslo, Oslo, Norway; ‡ Institute of Informatics, University of Oslo, Oslo, Norway

**Keywords:** Implantable cardioverter-defibrillator, Pacemaker, Transvenous lead extraction, Single sheath, High-volume center

## Abstract

**Background:**

Transvenous lead extraction (TLE) procedures can be complicated and are associated with a small but significant risk of cardiovascular complications. However, methods and tools vary among centers.

**Objective:**

The purpose of this study was to the present the methods and results of pacemaker and implantable cardioverter-defibrillator TLE procedures in our center over a 24-year period.

**Methods:**

From April 1997 through 2020, we attempted to extract 2964 leads in 1780 procedures and 1642 patients. We mainly utilized single sheath technique using snaring or mechanical rotational sheaths and steel sheaths when necessary. Difficult procedures were performed by an experienced cardiologist, and close supervision was emphasized. Most of the extractions were performed using local anesthesia with sedation.

**Results:**

Median age of patients was 65.0 [interquartile range 20.00] years, and median dwelling time of leads was 5.0 [7.0] years. Clinical success was achieved in 1739 procedures (97.7%) and complete technical success in 2841 leads (95.8%). Clinical success (leaving <4 cm of the lead in the body and achieving the clinical goal for the patient) was achieved for 79 leads (2.7%). TLE failed in 44 leads (1.1%) and 41 procedures (2.3%) among 36 patients (2.2%). There were 23 cases (1.3%) of major complications, with only 1 death directly related to the procedure (<0.1%). In addition, 2 patients with sepsis died within the first 24 hours after the procedure. No caval tears occurred.

**Conclusion:**

Single sheath lead extractions utilizing snaring or mechanical rotational sheaths were effective and safe in our high-volume center as performed by experienced operators.

**Key Findings**

▪ We present a single high-volume center experience with transvenous lead extraction over 24 years performed by experienced operators.
▪ We mainly utilized single sheath lead extraction using snaring or mechanical rotational sheaths when necessary.
▪ A clinical success rate of 97.7% with 1.3% major complications was achieved in 1780 extraction procedures.

## Introduction

The increasing number of implanted cardiac electronic devices in recent years has led to a growing number of patients requiring transvenous lead extraction (TLE).^1^^,^^2^ New indications for pacing, implantable cardioverter-defibrillator (ICD), and cardiac resynchronization therapy systems have increased the number of patients having a cardiac implantable electronic device (CIED) and consequently the number of CIED-related complications. In addition, the increased life expectancy of patients with a CIED is associated with an increased number of CIED generator replacements of or system change procedures for ICDs and cardiac resynchronization therapy devices.^3^^,^^4^

Although TLE techniques have evolved from simple traction to extraction using mechanical dilation and powered sheaths, percutaneous lead removal is still associated with small but significant procedural failure, morbidity, and mortality.^5^^,^^6^ This is mainly due to the presence of adhesions between leads and veins or cardiac chambers. We mainly used a single sheath (polypropylene) dilation technique combined with femoral and jugular snaring techniques, and the Cook Evolution^TM^ (Cook Medical, Bloomington, IN) or Spectranetics TightRail^TM^ (Spectranetics Corp. [now Philips], Colorado Springs, CO) mechanical rotational sheath (MRS).^7^^,^^8^ Our approach is similar to the techniques described by Bongiorni et al,^8^^,^^9^ with some modifications based on operator experience and preferences and on patient or lead characteristics.

The aim of this study was to describe our methods and techniques of TLE, as well as the success rate and rate of complications, in our high-volume center from 1997 through 2020.

## Methods

### Patient population and preparation

All consecutive patients admitted to Oslo University Hospital of Ullevaal for TLE from April 1997 to the end of 2020 were prospectively enrolled into a local TLE registry. There was no bias because no patients were referred for surgical extraction as the first option. The patients accepted for TLE were examined and prepared according to current consensus, including clinical examination, thorough device examination, blood tests, and thoracic radiography.^10^, ^11^, ^12^, ^13^ Preoperative transthoracic echocardiography was performed in all cases by experienced cardiologists, with special focus on the lead course through the tricuspid valve. If echocardiography indicated that 1 or more leads could be adherent to the tricuspid valve, the indication for TLE was re-evaluated (ie, was the indication for TLE definitive). If TLE was performed, close attention was paid in order to avoid valve damage (eg, avoiding excessive pulling of the lead). Transesophageal echocardiography was performed before TLE only in selected cases, mostly if lead endocarditis was suspected but was not obvious, or if left-sided valve endocarditis was suspected.

Anticoagulation and antiplatelet drugs were managed based on a risk-to-benefit analysis. The standard approach was to stop anticoagulation 2 days before the procedure. In cases of mechanical heart valves, the procedure was performed with patients having an international normalized ratio in the therapeutic range.

### Extraction procedure

The TLE procedures were performed in a hybrid laboratory with a cardiothoracic surgery team available within few minutes; a few cases were performed on-site in the operating room due to risk stratification. Patients were in the fasting state. All patients were monitored with blood pressure and heart rate monitoring, application of cutaneous pads for defibrillation, and invasive arterial blood pressure measuring in selected cases. Transvenous temporary pacing was used in pacemaker-dependent patients and in expected difficult cases. In cases with CIED infection, we tested whether discontinuation of beta-blocker and administration of theophylline or isoproterenol could increase heart rate to a hemodynamically sufficient level. This was performed to avoid insertion of unnecessary temporary leads and minimize the risk of reinfection of the new CIED on the contralateral side. Antibiotics were administered intravenously before the procedure in all cases.

The extraction procedures were performed by 7 cardiologists; 4 of whom had performed >250 TLE procedures each by the end of the 24-year period. The procedures were performed either by an experienced cardiologist or by a less experienced operator with an experienced cardiologist present in the laboratory. By the end of 2020, the least experienced cardiologist had performed 40 procedures, and the most experienced cardiologist had performed >700 lead TLE procedures. The patients were mostly sedated, and the procedures were performed with local anesthesia. General anesthesia was administered in selected cases only, with decisive factors being patient age, comorbidity, level of anxiety, mental state, and number, type, tip position. and age of the leads. Some patients were converted from local anesthesia and sedation to general anesthesia during the procedure because of complications or pain.

In most of our cases, the primary approach was the single sheath dilation technique as described by Bongiorni et al,^8^ customized to our experience and preferences. We cut the leads 6–8 cm from the venous entry site and used a locking stylet (Cook or Spectranetics) in most cases as opposed to Bongiorni et al, who used a regular stylet in their primary approach. The securing of the lead was sufficient in some cases to remove the lead with traction. If traction failed, we continued with the single sheath dilation technique. The single sheath dilation technique was used in the presence of exposed leads through the subclavian, cephalic, or jugular vein. Polypropylene sheaths (Cook or Spectranetics) were used. Most leads were extracted using 8.5F to 11.5F sheaths; in some cases, 13F sheaths were used. In the St. Jude Medical Riata ICD lead cases, a larger sheath was selected due to externalization. In accordance with the technique described by Bongiorni et al,^8^ we changed to a larger sheath diameter when resistance was met and often switched back and forth between sheath sizes to overcome areas of strong adhesions. If it was not possible to pass one of the adhesions along the lead, we changed to an MRS or snaring technique with transfemoral access (TFA) or internal transjugular access (ITA). When the lead was free-floating (ie, with no attachment to the entry site) from the beginning or the lead broke during the procedure, the TFA or ITA approach as described by Bongiorni et al was used.^9^

When the lead had grown into the clavicle, we used a steel sheath or the Cook Evolution Shortie^TM^ sheath. Procedural time was registered from skin incision to wound closure ("skin to skin") and included reimplant time in cases in which it was performed in the same procedure. Sheath time was defined as the time the sheaths were being rotated or otherwise used actively to advance along the lead. If for some reason the sheath was left inside the patient for a short time while tracking out another lead or implanting a new lead, this time was not included in sheath time. Since 2010, all patients were examined immediately after the extraction procedure in the laboratory or, in some cases, in the cardiac intensive care unit using hand-held echocardiography (Vscan^TM^, Horten, Norway) to evaluate for pericardial effusion; this was performed based on clinical suspicion before 2010. Damage to the tricuspid valve was registered, although not actively examined for in all patients. If cardiac complications were suspected or after difficult procedures, complete transthoracic echocardiographic examination was performed. The extraction procedure remained mostly unchanged during the 24-year period, except for increased use of MRS and anesthesia, especially in younger patients during recent years.

### Outcomes and definitions

The primary outcome of our study was success rate as a measure of efficacy. The primary safety outcome was complication rate during hospital stay and up to 30 days after.

Procedural outcomes were defined according to radiological outcome, with complete success defined as removal of the entire lead. Partial success was defined as removal of all of the lead except the distal 4 cm. Failure was defined as leaving a larger fragment, total failure of lead removal, or stopping the procedure because of a major complication.

Complications that occurred during TLE procedures or up to 30 days after were registered consecutively and according to the classification published in the North American Society for Pacing and Electrophysiology (NASPE) Policy Statement (Supplemental Table S1).^8^ If the category to which the complications should be assigned was in doubt, the more serious category was chosen. If a valve injury indicated surgery but contraindications led to observation, the complication was categorized as major. We did not have permission to collect long-term follow up data. Only the most important complication in each patient was registered systematically.

### Statistical analysis

Continuous variables are given as mean ± SD or median [interquartile range] according to data type and distribution. Kendall rank test was used to assess correlation (trend) between patient and procedural characteristics and ordinal categories of outcome. The Student *t* test, Wilcoxon rank-sum test, or Fisher exact test was used according to data type and distribution to test differences in variables between 2 groups.

Logistic regression analyses were used to assess the relationship between characteristics of patients, procedures, and outcome. Possible predictors were tested by univariate analysis, and significant predictors underwent multivariate logistic regression analyses with subsequent backward stepwise elimination of nonsignificant variables. Continuous variables with skewed distributions were divided into intervals to ease presentation of data, but sensitivity analyses were performed for all variables with skewed distribution after logarithmic transformation to ensure that results were in a similar range. If results were different, logarithmic transformation of the skewed variable was chosen for regression analysis. The Fisher exact test was used for categorical data to validate results from regression analysis because of the small number of cases in some categories of outcome. *P* ≤.05 was considered significant for all tests. Analyses were performed using JMP 9 software (SAS Institute, Cary, NC).

### Ethics

The local lead extraction registry was established with permission from the hospital data protection officer. Permission to publish anonymous data was given in September 2020 by the privacy officer on behalf of the hospital administration. The research reported in this paper adhered to the Helsinki Declaration

## Results

**Figure 1:**
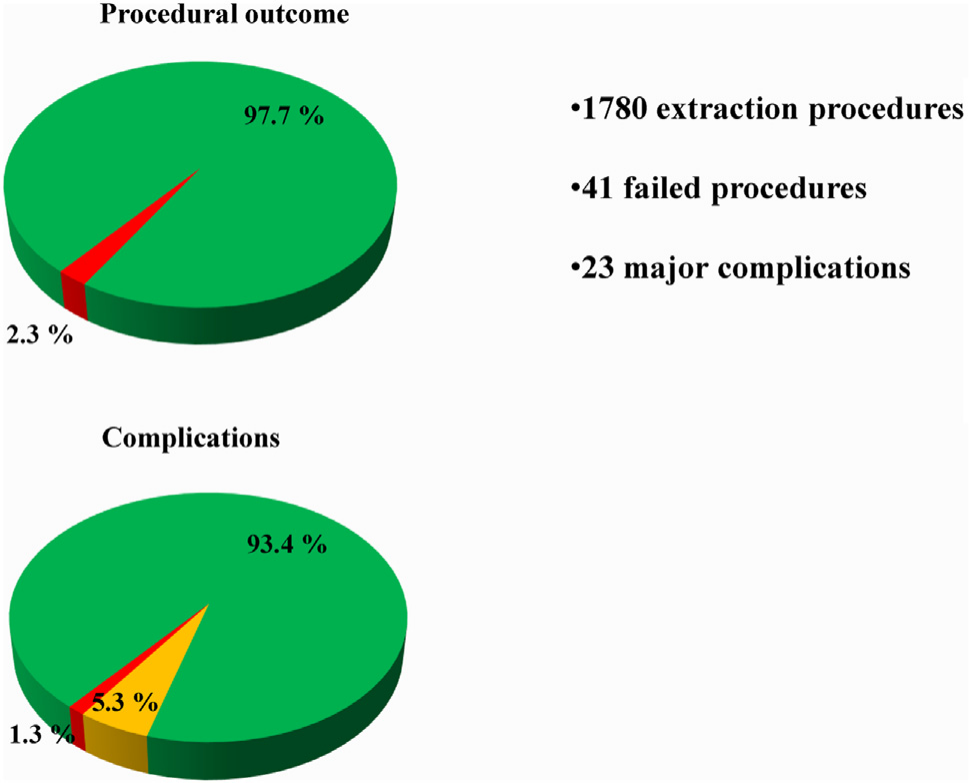
Main results in percentage of total number of transvenous lead extraction procedures. For procedural outcome, clinical success is shown in *green* and failure in *red.* For complications, major complications shown in *red,* minor or observation in *yellow,* and no complication in *green.*

**Figure 2:**
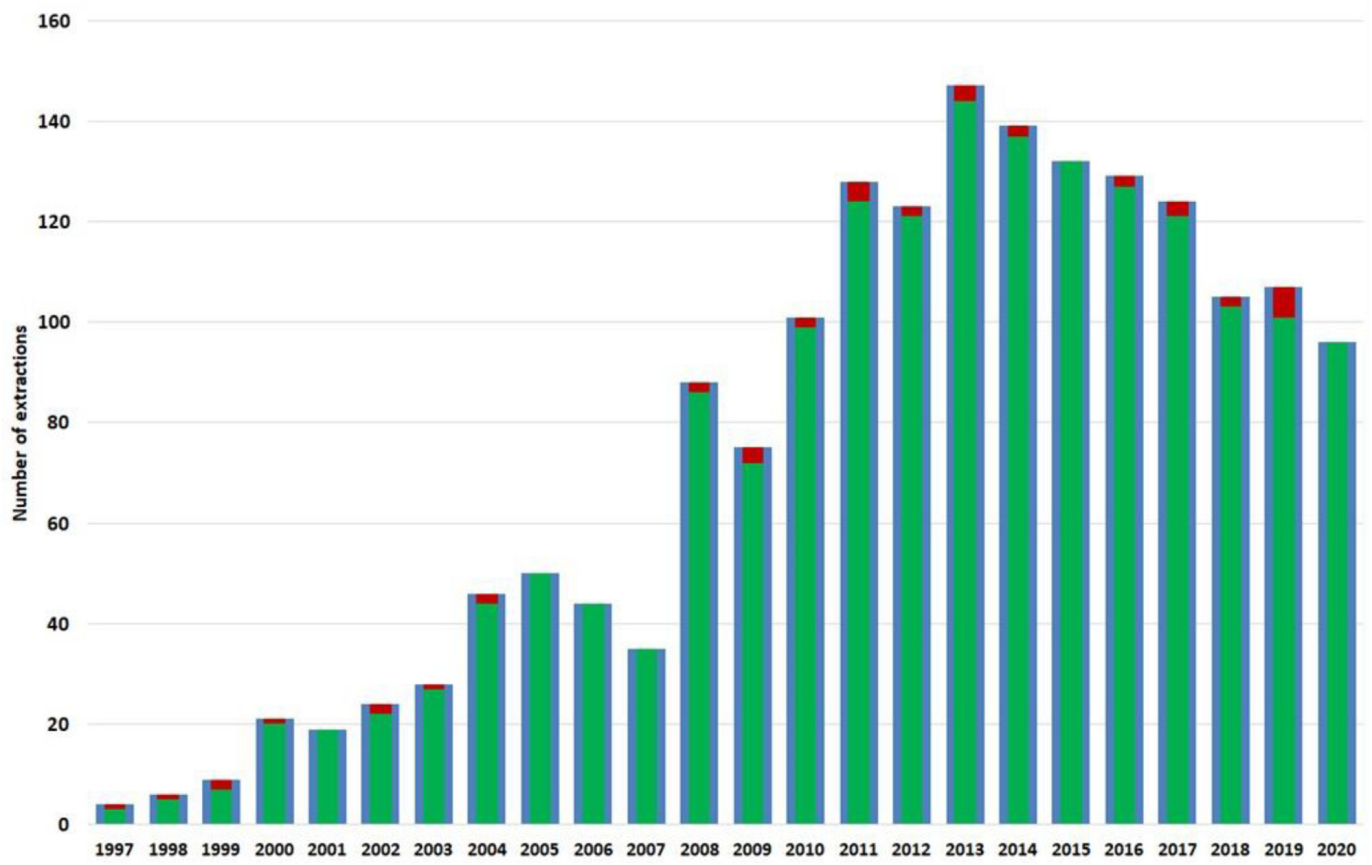
Number of transvenous lead extraction procedures each year. Total number shown in bars with *blue borders.* Number of transvenous lead extractions with achieved clinical success shown in *green bars* and number of failed transvenous lead extractions in *red bars.*

Between April 1997 and the end of 2020, 1780 procedures were performed in 1642 patients. Of the 1642 patients, 120 had >1 TLE procedure. Because some patients underwent repeat procedures due to failed cases, the 1780 procedures represent 1768 unique patient cases. Main results are shown in Figure 1. Main characteristics of patients, procedures, and leads are given in Table 1 and additionally according to level of technical success in Table 2. Further details are given in Supplemental Tables S2, S3, and S4. Number of procedures, procedures with clinical success, and failures per year are shown in Figure 2. Median age of the patients was 65.0 [20.00] years, and two-thirds were male. Infection was the predominant indication during the first 5 years, reached a minimum of 27.9% in 2013, and accounted for 47.4% of TLE indications from 2016 throughout 2020. Manual traction alone was used in 307 procedures (17.2%), and 765 leads (25.8%) were removed with manual traction alone. Median lead dwelling time was 5.0 [7.0] years; 685 leads had dwelling time >10 years, and 101 leads had dwelling time >20 years. Median procedural time was 70 [63] minutes.

**Table 1:** Patient, procedure, and lead characteristics

	
Age (y)	65 [20]
Male	1200 (67.4)
Age at implant of oldest lead (y)	58 [22.5]
Primary cardiac disease∗	
Primary electrical disease†	819 (46.0)
Coronary artery disease	496 ( 27.9)
Dilated cardiomyopathy	203 (11.4
Valvular heart disease	126 (7.1)
Congenital heart disease	100 (5.6)
Hypertrophic cardiomyopathy	31 (1.7)
Unclassified or no known heart disease	5 (0.3)
Indication	
Sepsis/lead endocarditis	253 (14.2)
Pocket infection	504 (28.4)
Noninfection‡	1022 (57.4)
Left ventricular ejection fraction§	
>50%	845 (47.5)
30%–50%	490 (27.5)
<30%	222 (12.5)
Unclassified or missing	223 (12.5)
Lead characteristics	
Lead type, n (%)	
Pacing leads	2085 (70.3)
ICD leads	703 (23.7)
Left ventricle–coronary sinus	132 (4.5)
VDD	32 (1.1)
SVC/array	12 (0.4)
Fixation type, n (%)	
Active fixation, screw	1913 (64.5)
Active fixation, tines	849 (28.6)
Passive fixation, unknown, or other	202 (6.8)
Tip location, n (%)	
Right atrium	1057 (35.7)
Right ventricle	1739 (58.7)
Coronary sinus and other	168 (5.7)
Dwelling time (y)	
Mean ± SD	6.6 ± 5.4
Median [interquartile range]	5.0 [7.0]

Total no. of procedures = 1780. Total no. of extracted leads = 2964.

Values are given as median [interquartile range] or n (%) unless otherwise indicated.

ICD = implantable cardioverter-defibrillator; SVC = superior vena cava.

∗ Most important cardiac disease. Only the primary cardiac disease was registered for each individual.

† Includes both conduction system disease and arrhythmia.

‡ Includes both absolute indications (eg, occluded vein and need for venous access), the considered best clinical option, and relative indications (eg, prophylactic extraction of recall leads, to avoid abandoned leads when system change or nonfunctioning lead).

§ Registered at first lead extraction procedure of each individual, not updated at repeated procedures.

**Table 2:** Procedure and lead characteristics according to level of technical success

No. of procedures (N = 1780)	Complete success	Partial success	Failure	*P* value
	1669	70	41	
Age (y)	65.0 [19.0]	65.5 [20.8]	46.0 [37.0]	.0282
Male	1136 (68.1)	45 (64.3)	19 (46.3)	.0201
Infection	705 (42.2)	36 (51.4)	17 (41.5)	NS
Dwelling time oldest lead (y)	5.0 [6.0]	8.5 [8.5]	12 [13.5]	<.0001
Procedural time (min)∗	67.0 [60.0]	108 [101.5]	152.5 [126.5]	<.0001
General anesthesia	123 (7.4)	8 (11.4)	10 (24.4)	.0006
Mechanical rotational sheaths†	264 (15.8)	15 (21.4)	15 (36.6)	<.0001
Snare†	32 (1.9)	13 (18.6)	11 (26.8)	<.0001
Major complication [n (% of procedures)]	15 (0.9)	5 (7.1)	3 (7.3)	<.0001
No. of leads to be removed (N = 2964)	2752	142	70	
No. of leads completely removed	2752 (100.0)	63 (44.4)	26 (37.1)	<.0001

Values are given as n, median [interquartile range], or n (%) unless otherwise indicated. *P* value by Kendall rank test.

∗ n = 1776 (4 missing). Time from skin incision to wound closure ("skin to skin"), including reimplant when performed in the same procedure.

† No. (%) of procedures in which this tool was used on at least 1 lead.

Clinical success (complete or partial technical success) was achieved in 1739 procedures (97.7%) (Table 2). Clinical success was achieved in repeated procedures in 5 patients who had failure at the first procedure; thus, clinical success was achieved in 1732 of 1768 cases (98.0%).

**Figure 3:**
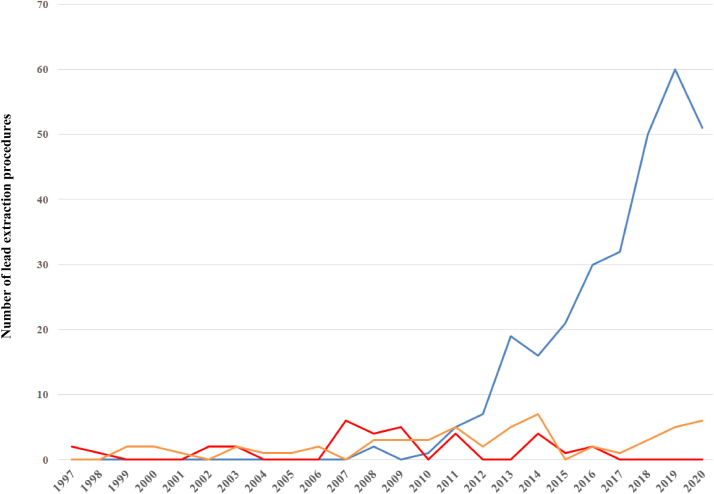
Number of transvenous lead extraction procedures each year involving specific extraction tools. Mechanical sheaths—mechanical rotating dilator sheath (Cook Evolution or Spectranetics TightRail)—are shown in *blue.* Steel sheaths shown in *red* and snaring in *green.* Polypropylene and locking stylets were used in most procedures and are not shown.

MRS were used since 2008 in 294 procedures (16.5%), with increasing use in recent years (Figure 3). The snaring technique was used in 56 TLE procedures (3.1%), with clinical success in 45 procedures (80.4%) (Table 2). TFA was used in 38 procedures, ITA in 21 procedures, and the 2 techniques combined in 15 procedures. Of the 12 procedures in which snaring from venous entry site was applied, only 1 was combined with TFA.

General anesthesia was given in <8% of the cases (Table 2 and Supplemental Table S5). These patients were younger, had older leads, and had more complex CIED history. Further characteristics according to method of anesthesia are given in Supplemental Table S5. A cardiothoracic surgery team was present on-site from the start of the procedure in 8 TLE procedures only.

Younger age, female sex, dwelling time of oldest lead, use of steel sheath, and longer x-ray time were associated with procedural failure in multivariate analysis (Supplemental Table S6).

The type of lead (ICD or pacemaker leads) was not associated with failure or with major complications (Supplemental Tables S6 and S7). ICD leads had a mean dwelling time of 5.0 [4.0] years, similar to other leads (5.0 [8.0] years).

One ICD lead subsequently was removed successfully by open heart surgery in a younger patient because of a free-floating end and the potential risk of ventricular arrhythmias. Another ICD lead fragment later was removed successfully by open heart surgery because of infection. Success rates and complication rates did not significantly vary with the level of operator experience (data not shown).

### Complications

A total of 23 major complications (1.3% of all procedures) occurred over a period of 24 years, with only 1 death directly related to the TLE procedure (0.06% mortality) and no caval tears (Table 3). Two patients died from sepsis within 24 hours after lead extraction, and 1 patient died of renal failure and sepsis within 1 week after TLE, for a total of 4 (0.23%) in-hospital (while staying at our hospital after TLE) deaths. Of 11 patients who required open heart surgery (Table 3), 4 underwent immediate surgery because of cardiac tamponade (no patients with tamponade required open heart surgery between 2008 and the end of 2020.) The most common major complication in recent years has been damage to the tricuspid valve. In total, 14 cases of tricuspid valve injury were registered, of which 7 were considered a major complication (Table 3). Some were not recognized immediately after the procedure but determined based on clinical suspicion of murmur or other indication for echocardiography later in the course. Of the patients with tricuspid valve injury initially categorized as observation, 1 had deterioration of heart failure secondary to damage of the valve and subsequently underwent a heart transplant months later. Thirty-two complications were classified as minor (1.8% of procedures) and 62 as observation (3.5%) (Table 4). However, all minor complications and observations may not have been registered, especially if they occurred after discharge from our hospital. Detailed information about patient, procedure, and lead characteristics according to complications are given in Supplemental Tables S8, S9, and S10. Female sex, dwelling time of oldest lead, and longer procedural time were associated with major complications in multivariate analysis (Supplemental Table S7).

**Table 3:** Major complications

Patient	Year	Oldest lead (y)	Indication	Complication	Treatment	Outcome
1	1999	23	Sepsis	Tamponade	Drainage, open heart surgery next day	Recovery
2	2002	15	Pocket infection	Tamponade	Immediate open heart surgery	Death
3	2004	2	Pocket infection	Respiratory/cardiac arrest	CPR	Recovery
4	2004	18	Pocket infection	Tamponade	Immediate open heart surgery	Recovery
5	2004	22	Noninfection	Tamponade	Immediate open heart surgery	Recovery
6	2005	8	Noninfection	Tamponade	Drainage	Recovery
7	2008	3	Noninfection	Bronchospasm	Intubation and inhalations	Recovery
8	2008	16	Noninfection	Tamponade	Immediate open heart surgery	Recovery
9	2009	17	Pocket infection	Valve injury	Observation	Minimal symptoms
10	2009	23	Noninfection	Valve injury	Open heart surgery, TVR	Recovery
11	2010	3	Pocket infection	Pneumothorax and hemothorax	Chest tube and transfusion	Recovery
12	2011	29	Pocket infection	Tamponade	Pericardial drainage	Recovery∗
13	2011	4	Noninfection	Respiratory/cardiac arrest	CPR	Recovery
14	2012	8	Pocket infection	Valve injury	Open heart surgery, valve repair	Recovery
15	2012	5	Noninfection	Valve injury	Open heart surgery, valve repair	Recovery
16	2014	11	Noninfection	Valve injury†	Open heart surgery, valve repair	Recovery
17	2014	10	Noninfection	Valve injury	Open heart surgery, TVR‡	Recovery
18	2015	9	Noninfection	Valve injury	Open heart surgery, valve repair	Recovery
19	2015	2	Noninfection	Pneumothorax	Chest tube	Recovery
20	2015	6	Sepsis	Tamponade	Pericardial drainage	Recovery
21	2016	6	Noninfection	Stroke	Cerebral decompression and rehabilitation	Hemiparesis
22	2016	4	Sepsis	Stroke/TIA	Examination, observation	Recovery
23	2020	4	Pocket infection	Thoracic bleeding	Transfusion and observation	Recovery

CPR = cardiopulmonary resuscitation; TVR = tricuspid valve regurgitation.

∗ Transient renal failure,

† Detected 2 months after lead extraction because of gradually increasing dyspnea.

‡ Detected 1 week after lead extraction.

**Table 4:** Minor and observational complications

Minor complications (N = 32)	
Pericardial effusion not requiring pericardiocentesis or surgical intervention	3
Hemodynamically significant air embolism	0
Pulmonary embolism not requiring intervention	3
Vascular repair near the implant site or venous entry site	0
Arrhythmia requiring cardioversion/new conduction block and arrest requiring pacing	6
Hematoma at pocket site requiring drainage	10
Arm swelling or thrombosis of implant veins resulting in medical intervention	9
Sepsis in a previously nonseptic patient with infection	1
Pacing system related infection of a previously noninfected site	0
Observation (N = 62)	
Transient hypotension that responds to fluids or minor pharmacologic intervention	17
Nonsignificant air embolism	0
Small pneumothorax not requiring intervention	2
Ectopy not requiring cardioversion∗	4
Arm swelling or thrombosis of implant veins without need for medical intervention∗	2
Pain at cutdown site∗	0
Myocardial avulsion without sequelae†	7
Migrated lead fragment without sequelae	1
Hematoma not requiring drainage	29

Values are given as no. of procedures during/after which a particular complication occurred.

∗ Less systematic registration.

† All of these were injuries to the tricuspid valve.

## Discussion

We report the results of CIED lead extractions in 1780 patients at our high-volume center. Clinical success was achieved in 1739 procedures (97.7%). The rate of major complications was 1.3%, and mortality rate was 0.06%. Patients included in our registry were of similar age as in other TLE reports, and approximately two-thirds were male, as also reported in other publications.^14^ Less than half of the procedures performed in our center were performed because of infection. This is somewhat lower than in the ELECTRA (European Lead Extraction Controlled) registry^6^ but similar to the results of Brunner et al.^15^ Because we lack data on blood tests and comorbidity and have incomplete registration of left ventricular ejection fraction and New York Heart Association functional class, a more exact comparison between our population and other populations undergoing TLE would be inaccurate.

Our procedural success rates are in line with results from other high-volume centers.^3^^,^^6^^,^^8^^,^^15^^,^^16^ We had relatively conservative use of resources because general anesthesia was confined to selected cases comprising <8% of TLE procedures. Furthermore, an on-site cardiothoracic surgery team and per operative echocardiography were used for exceptional cases only. We believe that our approach and use of resources are cost-effective, although mainly using unpowered sheaths may increase procedural and fluoroscopy times. However, our procedural times were shorter than those in the ELECTRA study^6^ and the study of Brunner et al,^15^ but we lack exact data on costs.

In accordance with findings from other patient series, procedural failure was independently associated with young age and dwelling time of leads, probably due to more fibrotic adhesions around the leads, often also calcified.^14^ Furthermore, female sex is known to increase the risk of complications and was independently associated with both procedural failure and major complications in accordance with results from other studies.^3^^,^^16^^,^^17^

It seems that we had a higher threshold of converting to snaring and a TFA or ITA approach than Bongiorni et al.^9^ More frequent use of sheath techniques with snaring and a TFA and ITA approach may have increased our success rates.^9^ Somewhat surprisingly, we did not find significant associations between success rates according to the main operator’s level of experience. We believe this may be explained by two factors. First, the most experienced operator was always available on-site and could aid the less experienced colleague if necessary. Second, the presumed difficult cases generally were assigned to the more experienced cardiologist.

Only 1 death directly related to the procedure and no caval tears occurred during the 24-year period. These results seem to be similar to, or even lower than, those reported in other registries and studies. Furthermore, the rate of other major complications was in a similar range as reported in other studies.^3^^,^^6^^,^^8^^,^^12^^,^^16^ Potential differences in patient characteristics may explain minor differences in outcomes.

Although all complications were systematically and consecutively registered during the stay in our hospitals, we cannot preclude that the rates of some complications may have been higher if systematic and targeted examinations for these complications had been undertaken.

With increasing experience and better tools has come a reduction in the number of cases with cardiac tamponade.^5^^,^^6^ In recent years, we registered some cases of damage to the tricuspid valve. Whether using MRS more and earlier in the procedure could reduce the incidence of valvular damages seems to be an unresolved question.^18^

Our success and complication rates are comparable to those of other high-volume centers performing pacemaker and ICD lead extractions.^5^^,^^6^

Several factors may contribute to the relatively low complication rate in our center. One is careful use of the single sheath technique as the primary TLE approach. Performance of procedures at a high-volume center, careful patient examination and evaluation, meticulous focus on reducing complications by all means possible, and close supervision and guidance from experienced TLE cardiologists are likely to prevent complications. A relatively benign patient profile (mostly men), a high proportion of active fixation leads, and a relatively low mean dwelling time also may have influenced our results. However, we had a significant proportion of leads with dwelling time >10 years. Recently the MRS Cook Evolution and Spectranetics TightRail sheaths have become important supplemental extraction tools.^19^, ^20^, ^21^ Because these sheaths are more “aggressive” and effective than regular polypropylene or metal sheaths, concern has been raised regarding their safety. Therefore, these tools still are not the first option at our center. Importantly, to reduce the risk of cardiac tear, the procedure has been switched back to use of polypropylene sheaths after passing an adherence, if possible. However, the use of MRS have increased since 2012 year at our center, and especially since 2017. Importantly, we used the PinVise^TM^ (Cook Medical) for improving grip and rotation on dilator sheaths until the PinVise was no longer available on the market after CE approval was not renewed in August 2017. This rotational tool has not yet been replaced by any available tool of the same quality. MRS have been more available, and there is increasing documentation on the safety and efficacy of MRS, hence the change in policy.

### Resources and prevention of serious complications

Many experts on CIED lead extractions today strongly advocate the use of intraprocedural transesophageal echocardiography. Another recommendation is placement of a guidewire from the groin to the superior caval vein to enable immediate use of a caval occluder balloon in case of a caval tear. Because we had observed no caval tears in 1780 procedures, we believe that placing a wire from the groin to the superior vena cava is not necessary in general. In many centers, lead extractions are also performed with general anesthesia with thoracic surgeons on-site. These preventive measures are costly, and we have achieved similar success and safety rates without these measures.

### Study strengths and limitations

All patients requiring a pacemaker or ICD lead extraction in our center have been registered consecutively, and the data are complete for most parameters presented in this study for almost all patients.

Registration of comorbidities has been less complete, and the registration of left ventricular function is missing for many patients. Furthermore, left ventricular ejection fraction was registered for patients before their first extraction procedure and was not updated for repeated TLE. Blood tests and medications were been registered. Data on chosen method of anesthesia, steel sheaths, transesophageal echocardiography, and cardiothoracic surgeon backup on-site were registered retrospectively.

At the beginning of the transvenous extraction era at our hospital, the main focus was cardiac tamponade as the major complication; hence, it is possible that some cases of asymptomatic tricuspid valve regurgitation were missed. In addition, suboptimal quality of echocardiographic method/tools (hand-held echocardiographic scanner [Vscan] in many cases) may have underestimated the incidence of tricuspid valve damage.

The most important limitation is the lack of systematic registration of outcome after the initial hospital admission because of the lack of permission to do so from the Norwegian legislation. Therefore, late complications, especially minor ones, may be missed. Furthermore, only the most serious complication for each patient was registered systematically.

## Conclusion

Single sheath lead extractions using snaring sheaths or MRS were effective and safe in our high-volume center as performed by experienced operators.
