# Supplementary material for: Transvenous lead extractions in a single high-volume center over a 24-year period: High success rate and low complication rate
Source: Heart Rhythm O2. 2023 Jan 20;4(4):232–40. doi: 10.1016/j.hroo.2023.01.003 (PMC10134393; doi:10.1016/j.hroo.2023.01.003)
Supplement: Supplemental Tables [file mmc1.docx]

| **Table S1 Classification of complications according to NASPE Policy Statement 2000**† |
| --- |
| **1. Major complication** |
| 1.Death‡ |
| 2.Cardiac avulsion or tear requiring thoracotomy, periocardiosentesis, chest tube or surgical repair |
| 3.Vascular avulsion or tear requiring thoracotomy, periocardiosentesis, chest tube or surgical repair |
| 4. Hemothorax or severe bleeding from any source requiring transfusion |
| 5. Pneumothorax requiring chest tube drainage |
| 6. Pulmonary embolism requiring surgical intervention |
| 7. Respiratory arrest |
| 8. Septic shock |
| 9. Stroke |
| **2. Minor complication** |
| 1. Pericardial effusion not requiring pericardiocentesis or surgical intervention |
| 2. Haemodynamically significant air embolism |
| 3. Pulmonary embolism not requiring interventionn |
| 4. Vascular repair near the implant site or venous entry site |
| 5. Arrhytmia requiring cardioversion/new conduction block and arrest requiring pacing |
| 6. Haematoma at the pocket site requiring drainage |
| 7. Arm swelling or trombosis of implant veins resulting in medical intervention |
| 8. Sepsis in a previously non-septic patient with infection |
| 9. Pacing system related infection of a previously non-infected site |
| **3. Observation** |
| 1. Transient hypotension that responds to fluids or minor pharmacologic intervention |
| 2. Non-significant air embolism |
| 3. Small pneumothorax not requiring intervention |
| 4. Ectopy not requiring cardioversion |
| 5. Arm swelling or trombosis of implant veins without need for medical intervention |
| 6. Pain at the cut-down site |
| 7. Myocardial avulsion without sequelae |
| 8. Migrated lead fragment without sequealae |
| 9. Haematoma not requiring drainage |
| †.Love CJ, Wilkoff BL, Byrd CL, Belott PH, Brinker JA, Fearnot NE, et al. Recommendations for extraction of chronically implanted transvenous pacing and defibrillator leads: indications, facilities, training. North American Society of Pacing and Electrophysiology Lead Extraction Conference Faculty. Pacing Clin Electrophysiol 2000;23:544-51.  ‡ Death from multisystem or associated patient conditions would be included if procedure caused or contributed to the final event.  ‡Death clearly unrelated to the procedure would not be included, for example death in a septic patient who had a perforation and could not be resuscitated would be included. Death in a septic patient who underwent lead extraction and later died of sepsis would not. |

| **Table S2 Patient characteristics according to level of technical success** | | | | | | | | | | | | |  |  |
| --- | --- | --- | --- | --- | --- | --- | --- | --- | --- | --- | --- | --- | --- | --- |
| **Outcome** | **Complete success** | | | **Partial success** | | | **Failure** | | | ***P*** | | |  |  |
| **Procedures, n = 1780** | **n = 1669** | | | **n = 70** | | | **n = 41** | | |  | | |  |  |
| Age years, median (IQR) | 65.0 (19.0) | | | 65.5 (20.8) | | | 46.0 (37.0) | | | 0.0282 | | |  |  |
| Male, n (%) | 1136 (68.1) | | | 45 (64.3) | | | 19 (46.3) | | | 0.0201 | | |  |  |
| Left ventricular ejection fraction ≥ 40%, n (%)† | 988 (59.2) | | | 45 (64.3) | | | 29 (70.7) | | | ns | | |  |  |
| Left ventricular ejection fraction < 40%, n (%) | 481 (28.8) | | | 7 (10.0) | | | 7 (17.1) | | | ns | | |  |  |
| Left ventricular ejection fraction not registered, n (%) | 200 (12.0) | | | 18 (25.7) | | | 5 (12.2) | | | na | | |  |  |
| NYHA class I, n (%) | 818 (49.0) | | | 42 (60.0) | | | 25 (61.0) | | | 0.0271 | | |  |  |
| NYHA class II, n (%) | 296 (17.7) | | | 9 (12,9) | | | 6 (14.6) | | | ns | | |  |  |
| NYHA class III or IV, n (%) | 234 (14.0) | | | 6 (8.6) | | | 2 (4.9) | | | 0.0410 | | |  |  |
| NYHA class unknown or missing, n (%) | 321 (19.2) | | | 13 (18.6) | | | 8 (19.5) | | | na | | |  |  |
| Primary indication for CIED† |  | | |  | | |  | | |  | | |  |  |
| Primary electrical disease‡, n (%) | 761 (45.6) | | | 39 (55.7) | | | 19 (46.3) | | | ns | | |  |  |
| Coronary artery disease, n (%) | 471 (28.2) | | | 16 (22.9) | | | 9 (22.0) | | | ns | | |  |  |
| Dilated cardiomyopathy, n (%) | 191 (11.4) | | | 5 (7.1) | | | 7 (17.1) | | | ns | | |  |  |
| Valvular heart disease, n (%) | 119 (7.1) | | | 6 (8.6) | | | 1 (2.4) | | | ns | | |  |  |
| Congenital heart disease, n (%) | 91 (5.5) | | | 4 (5.7) | | | 5 (12.2) | | | ns | | |  |  |
| Hypertrophic cardiomyopathy, n (%) | 31 (1.9) | | | 0 (0.0) | | | 0 (0.0) | | | ns | | |  |  |
| Unclassified or no known heart disease, n (%) | 5 (0.3) | | | 0 (0.0) | | | 0 (0.0) | | | ns | | |  |  |
| † Only the most important cardiac diagnosis was registered ‡ Includes both conduction system disease and arrhythmia, ns = not significant, na = not aplicable. | | | | | | | | | | | | |  |  |
| **Table S3 Procedure and lead characteristics according to level of technical success** | | | | | | | | | | | | | | |
| **Outcome** | | | **Complete success** | | | **Partial success** | | | **Failure** | | | ***P*** | | |
| **Procedures, n = 1780** | | | **n = 1669** | | | **n = 70** | | | **n = 41** | | |  | | |
| Sepsis/lead endocarditis, n (%) | | | 239 (14.3) | | | 8 (11.4) | | | 6 (14.6) | | | ns | | |
| Pocket infection, n (%) | | | 466 (27.9) | | | 28 (40.0) | | | 11 (26.8) | | | ns | | |
| Non-infection, n (%) | | | 964 (57.8) | | | 34 (48.6) | | | 24 (58.5) | | | ns | | |
| Dwelling time oldest lead, years median (IQR) | | | 5.0 (6.0) | | | 8.5 (8.5) | | | 12 (13.5) | | | <0.0001 | | |
| Procedure time, minutes median (IQR)† | | | 67.0 (60.0) | | | 108 (101.5) | | | 152.5 (126.5) | | | <0.0001 | | |
| X-ray time , minutes, median (IQR)‡ | | | 8.0 (12.0) | | | 19.0 (30.0) | | | 33.0 (33.0) | | | <0.0001 | | |
| Major complication, n (% of procedures) | | | 15 (0.9) | | | 5 (7.1) | | | 3 (7.3) | | | <0.0001 | | |
| Steel sheath, n (% of procedures)§ | | | 26 (1.6) | | | 3 (4.3) | | | 4 (9.8) | | | 0.0003 | | |
| **Total number of leads to be removed, n = 2964** | | | **2752** | | | **142** | | | **70** | | |  | | |
| **Extraction tool , number of leads (% )**¶ | | |  | | |  | | |  | | |  | | |
| Lock wire, n (%) | | | 1879 (68.3) | | | 116 (81.7) | | | 57 (81.4) | | | <0.0001 | | |
| Single sheath, n (%) | | | 1883 (68.4) | | | 117 (82.4) | | | 58 (82.9) | | | <0.0001 | | |
| Mechanical rotational sheaths, n (%) | | | 385 (14.0) | | | 23 (16.2) | | | 24 (34.3) | | | 0.0007 | | |
| Snare, n (%) | | | 34 (1.2) | | | 14 (9.9) | | | 11 (15.7) | | | <0.0001 | | |
| Dual sheath, n (%) | | | 10 (0.4) | | | 0 (0.0) | | | 2 (3.0) | | | ns | | |
| Track alone, n (%)# | | | 747 (27.1) | | | 14 (9.9) | | | 4 (5.7) | | | <0.0001 | | |
| Sheath time, minutes median (IQR)#† | | | 3.0 (5.0) | | | 5.0 (10.5) | | | 10 (14.5) | | | <0.001 | | |
| †n = 1776, 4 missing. Time from skin incision to wound closure. ‡ n= 1742, 38 missing. § not registered for individual leads, but procedures. ¶ % of leads within each category of outcome # not on lead failure, but successfully removed leads in the failed procedure #† time of active use per lead for those leads extracted with sheath and/or mechanical sheath, n = 2014/2193 (95.9%) of leads were sheaths were used, ns = not significant | | | | | | | | | | | | | | |
| **Table S4 Procedure and lead characteristics according to level of technical success** | | | | | | | | | | | | | |  |
| **Outcome** | | **Complete success** | | | **Partial success** | | | **Failure** | | | ***P*** | | |  |
| **Procedures, n = 1780** | | **n = 1669** | | | **n = 70** | | | **n = 41** | | |  | | |  |
| **Total number of leads to be removed, n** | | **2752** | | | **142** | | | **70** | | |  | | |  |
| Number of leads to extract per procedure, mean (SD) | | 1.65 (0.68) | | | 2.03 (0.88) | | | 1.71 (0.72) | | | <0.0001 | | |  |
| Pacemakerleads, n (%) | | 191 (69.7) | | | 120 (84.5) | | | 47 (67.1) | | | 0.0070 | | |  |
| ICD-leads, n (%) | | 673 (24.5) | | | 12 (8.5) | | | 18 (25.7) | | | 0.0010 | | |  |
| Left ventricle/coronary sinus leads, n ( %) | | 121 (4.4) | | | 9 (6.3) | | | 2 (2.9) | | | ns | | |  |
| VDD-leads, n (%) | | 29 (1.1) | | | 1 (0.7) | | | 2 (2.9) | | | ns | | |  |
| SVC/Arrays, n (%) | | 11 (0.4) | | | 0 (0.0) | | | 1 (1.4) | | | ns | | |  |
| Leads with active fixation, screw, n (%) | | 1827 (66.4) | | | 57 (40.1) | | | 29 (41.4) | | | <0.0001 | | |  |
| Leads with active fixation, tine, n (%) | | 737 (26.8) | | | 75 (52.8) | | | 37 (52.9) | | | <0.0001 | | |  |
| Passive fixation, unknown or other, n (%) | | 188 (6.8) | | | 10 (7.0) | | | 4 (5.7) | | | ns | | |  |
| **Specific lead types, total number of leads, n** | | **2752** | | | **142** | | | **70** | | |  | | |  |
| Guidant 44-series, n (%) | | 291 (10.6) | | | 41 (28.9) | | | 3 (4.3) | | | <0.0001 | | |  |
| Medtronic Starfix, n (%) | | 6 (0.2) | | | 0 (0.0) | | | 0 (0.0) | | | ns | | |  |
| Medtronic Sprint Fidelis, n (%) | | 194 (7.0) | | | 1 (0.7) | | | 0 (0.0) | | | ns | | |  |
| St.Jude Riata, n (%) | | 22 (0.8) | | | 4 (2.8) | | | 0 (0.0) | | | ns | | |  |
| St.Jude Quickflex, n (%) | | 16 (0.6) | | | 0 (0.0) | | | 0 (0.0) | | | ns | | |  |
| Medtronic Select Secure, n (%) | | 15 (0.5) | | | 0 (0.0) | | | 0 (0.0) | | | ns | | |  |
| None of the specific types above, n (%) | | 2208 (80.2) | | | 96 (67.6) | | | 67 (95.7) | | | ns | | |  |
| (%) = % of leads within each category of outcome, ns = not significant | | | | | | | | | | | | | |  |

| **Table S5 Characteristics of patients and procedures according to method of anesthesia** | | | |
| --- | --- | --- | --- |
|  | **Sedation** | **General anesthesia** | *P* |
| **Characteristics** | **n = 1639** | **n =141** |  |
| Age, years median (IQR) | 66.0 (18.0) | 40.0 (36.5) | <0.0001 |
| Male gender, n (%) | 1141 (69.6) | 59 (41.8) | <0.0001 |
| Oldest lead to be removed, years median (IQR) | 5.0 (6.0) | 9.0 (8.5) | <0.0001 |
| Two or more CIED replacements, n (%) | 314 (19.2) | 55 (39.0) | <0.0001 |
| **Primary cardiac disease**† |  |  |  |
| Primary electrical disease‡, n (%) | 757 (46.2) | 62 (44.0) | ns |
| Coronary artery disease, n (%) | 484 (29.5) | 12 (8.5) | <0.0001 |
| Dilated cardiomyopathy, n (%) | 189 (11.5) | 14 (9.9) | ns |
| Valvular heart disease, n (%) | 118 (7.2) | 8 (5.7) | ns |
| Congenital heart disease, n (%) | 57 (3.5) | 43 (30.5) | <0.0001 |
| Hypertrophic cardiomyopathy, n (%) | 29 (1.8) | 2 (1.4) | ns |
| Unclassified heart disease, n (%) | 5 (0.3) | 0 (0.0) | ns |
| **Indication** |  |  |  |
| Sepsis/lead endocarditis, n (%) | 236 (14.4) | 17 (12.1) | ns |
| Pocket infection, n (%) | 486 (29.7) | 19 (13.5) | <0.0001 |
| Non-infection, n (%) | 917 (55.9) | 105 (74.5) | <0.0001 |
| **Left ventricular ejection fraction**§ |  |  |  |
| >50%, n (%) | 751 (45.8) | 94 (66.7) | <0.0001 |
| 30 – 50%, n (%) | 459 (28.0) | 31 (22.0) | ns |
| <30%, n (%) | 218 (13.3) | 4 (2.8) | <0.0001 |
| Unclassified or missing, n (%) | 211 (12.9) | 12 (8.5) |  |
| **Lead characteristics** |  |  |  |
| **Total, n** | **2726** | **238** |  |
| Pacing leads, n (%) | 1905 (69.9) | 180 (75.6) | ns |
| ICD leads, n (%) | 652 (23.9) | 51 (21.4) | ns |
| Left ventricle - coronary sinus, n (%) | 127 (4.7) | 5 (2.1) | ns |
| VDD, n (%) | 31 (1.1) | 1 (0.4) | ns |
| SVC/array | 11 (0.4) | 1 (0.4) | ns |
| **Dwelling time of leads, years** |  |  |  |
| Mean ± SD | 6.4 (5.3) | 9.7 (6.4) | <0.0001 |
| Median (IQR) | 5 (7.0) | 9 (8.3) |  |
| **Procedure characteristics** |  |  |  |
| Procedure time, minutes median (IQR)¶ | 68.0 (62.0) | 80.0 (69.0) | <0.0001 |
| **Extraction tool, n (% of procedures)**# |  |  |  |
| Locking stylet, n (%) | 1268 (77.4) | 128 (90.8) | <0.0001 |
| Single sheath, n (%) | 1281 (78.2) | 128 (90.8) | 0.0002 |
| Mechanical sheaths, n (%) | 245 (14.9) | 49 (34.8) | <0.0001 |
| Snare, n (%) | 43 (2.6) | 13 (9.2) | 0.0003 |
| Dual sheath, n (%) | 11 (0.7) | 0 (0.0) | ns |
| Traction alone, n (%) | 543 (33.1) | 23 (16.3) | <0.0001 |
| Steel sheath, n (%) | 27 (1.6) | 6 (4.3) | 0.0413 |
| Major complication, n (%) | 18 (1.1) | 5 (3.5) | 0.0305 |
| †Most important cardiac disease. Only the primary cardiac disease was registered for each individual. ‡Includes both conduction system disease and arrhythmia. IQR = Interquartile range § Registered at first lead extraction procedure of each individual, not updated at repeated procedures. ¶Time from skin incision to wound closure. Some patient were converted to general anesthesia because of complications. #Used on at least one lead in procedure. ns = not significant | | | |

| **Table S6 Patient and procedure characteristics as possible and significant predictors of procedure failure** | | | | |
| --- | --- | --- | --- | --- |
|  | **Univariate analysis** | | **Multivariate analysis** | |
|  | **OR (95% CI)** | ***P*** | **OR (95% CI)** | ***P*** |
| Age, per 1 SD increase | 0.54 (0.42 – 0.70) | <0.0001 | 0.64 (0.47 – 0.88) | 0.0056 |
| Female gender | 2.45 (1.31 – 4.61) | 0.0049 | 2.20 (1.08 – 4.57) | 0.0307 |
| CIED history, two or more replacements/revisions | 4.20 (2.24 – 7.88) | <0.0001 |  |  |
| Congenital heart disease yes/no | 2.40 (0.81 – 5.74) | ns |  |  |
| Infection | 0.95 (0.50 – 1.78) | ns |  |  |
| Left ventricular ejection fraction below 40%† | 0.78 (0.72 – 0.84) | <0.0001 |  |  |
| Dwelling time oldest lead per 5 years | 1.95 (1.60 –2.38) | <0.0001 | 1.76 (1.38 –2.25) | <0.0001 |
| Log number leads to be removed | 1.24 (0.36 – 4.17) | ns |  |  |
| ICD-lead yes/no | 1.02 (0.53 – 1.90) | ns |  |  |
| One or more tined lead(s) | 2.42 (1.30 – 4.62) | 0.0054 |  |  |
| Fishing, snare yes/no | 13.80 (6.28 – 28.60) | <0.0001 |  |  |
| Single sheath yes/no | 1.55 (0.70 – 4.12 ) | ns |  |  |
| Mechanical rotational sheath yes/no | 3.02 (1.54 – 5.70) | 0.017 |  |  |
| Steel sheath yes/no | 6.40 (1.8 – 17.2) | 0.0065 | 4.78 (1.03 – 16.23) | 0.0460 |
| Lock wire | 1.34 (0.63 – 3.33) | ns |  |  |
| Procedure time per 15 min increase | 1.26 (1.19 – 1.34) | <0.0001 |  |  |
| LogX-ray time | 4.01 (2.77 – 5.98) | <0.0001 | 3.42 (2.29 – 5.27) | <0.0001 |
| † Left ventricular ejection fraction was registered for 1557/1780 (87.5%) of procedures 36/41 with failure . ‡registered for 1776 procedures, 40/41 (97.6%) with failure. § registered for 1742 procedures, 39/41 (95.1%) with failure. ns = not significant | | | | |

| **Table S7 Patient and procedure characteristics of possible and significant predictors of major complications** | | | | |
| --- | --- | --- | --- | --- |
|  | **Univariate analysis** | | **Multivariate analysis** | |
|  | **OR (95% CI)** | ***p*** | **OR (95% CI)** | ***p*** |
| Age, per 1 SD | 0.88 (0.60 – 1.33) | ns |  |  |
| Female gender | 2.73 (1.19 – 6.43) | 0.0177 | 3.03 (1.30 – 7.28) | 0.0103 |
| CIED history, two or more replacements/revisions | 4.28 (1.86 – 9.93) | 0.0008 |  |  |
| Infection yes/no | 1.48 (0.64 – 3.42) | ns |  |  |
| Left ventricular ejection fraction below 40%† | 0.54 (0.16 – 1.45) | ns |  |  |
| Dwelling time oldest lead per 5 years | 1.67 (1.27 – 2.14) | 0.0005 | 1.44 (1.07 – 1.90) | 0.0174 |
| Log number of leads to be removed | 2.31 (0.85 – 6.49) | ns |  |  |
| ICD-lead yes/no | 0.69 (0.27 – 1.63) | ns |  |  |
| One or more tined lead(s) | 2.65 (1.16 – 6.39) | 0.0214 |  |  |
| Fishing, snare yes/no | 4.82 (1.11 – 14.6) | 0.0377 |  |  |
| Single sheath yes/no‡ | 6.19 (0.83 – 46.05) | ns |  |  |
| Mechanical rotational sheath yes/no | 1.06 (0.31 – 2.86) | ns |  |  |
| Steel sheath yes/no | 5.30 (0.83 – 19.2) | ns |  |  |
| Lock wire‡ | 6.04 (0.81 – 44.98) | ns |  |  |
| Procedure time, per 15 minutes increase§ | 1.15 (1.07 – 1.23) | 0.0005 | 1.12 (1.03 – 1.21 ) | 0.0087 |
| Log X-ray time¶ | 1.38 (0.92 - 2.09) | ns |  |  |
| ns = not significant. †Registered for only 1557/1780 procedures (87.5%), 19/23 (82.6%) with major complication ‡Only one procedure in major complication group not involving use of single sheath and one without lock wire. Fischer`s exact test applied for OR. § registered for 1776 procedures, but for all 23 with major complications ¶1742 procedures, and 22/23 (95.6%) with major complications, ns = not significant | | | |  |

| **Table S8 Patient characteristics according to complications** | | | | |
| --- | --- | --- | --- | --- |
| **Complications** | **None** | **Minor or observation** | **Major** | ***P*** |
| **Procedures, n = 1780** | **n = 1663** | **n = 94** | **n = 23** |  |
| Age years, median (IQR) | 65.0 (20.0) | 64.5 (19.5) | 65.0 (18.0) | ns |
| Male, n (%) | 1128 (67.8) | 62 (66.0) | 10 (43.5) | ns |
| Left ventricular ejection fraction ≥ 40%, n (%) | 993 (59.7) | 54 (57.4) | 15 (65.2) | <0.001 |
| Left ventricular ejection fraction < 40%, n (%) | 467 (28.1) | 23 (25.6) | 4 (17.4) | ns |
| Left ventricular ejection fraction not registered, n (%) | 204 (12.3) | 15 (16.7) | 4 (17.4) | ns |
| NYHA class I, n (%) | 822 (49.4) | 50 (53.2) | 13 (56.5) | ns |
| NYHA class II, n (%) | 296 (17.8) | 12 (12.8) | 3 (13.0) | ns |
| NYHA class III or IV, n (%) | 226 (13.6) | 14 (14.9) | 2 (8.7) | ns |
| NYHA class unknown or missing, n (%) | 319 (19.2) | 18 (19.1) | 5 (21.7) | ns |
| Primary indication for CIED |  |  |  |  |
| Primary electrical disease†, n (%) | 761 (45.8) | 42 (44.7) | 16 (69.6) | ns |
| Coronary artery disease, n (%) | 461 (27.7) | 30 (31.9) | 5 (21.7) | ns |
| Dilated cardiomyopathy, n (%) | 194 (11.7) | 9 (9.6) | 0 (0.0) | ns |
| Valvular heart disease, n (%) | 119 (7.2) | 6 (6.4) | 1 (4.3) | ns |
| Congenital heart disease, n (%) | 93 (5.6) | 6 (6.4) | 1 (4.3) | ns |
| Hypertrophic cardiomyopathy, n (%) | 30 (1.8) | 1 (1.1) | 0 (0.0) | ns |
| Unclassified or no known heart disease, n (%) | 5 (0.3) | 0 (0.0) | 0 (0.0) | ns |
| †Includes both conduction system disease and arrhythmia | | | | |

| **Table S9 Procedure and lead characteristics according to complications** | | | | |
| --- | --- | --- | --- | --- |
| **Complications** | **None** | **Minor or observation** | **Major** | ***P*** |
| **Procedures, n = 1780** | **n = 1663** | **n = 94** | **n = 23** |  |
| **Total number of leads to be removed, n = 2964** | **2749** | **170** | **45** |  |
| Number of leads to extract per procedure, mean (SD) | 1.65 (0.69) | 1.81 (0.74) | 1.96 (0.93) | 0.0122 |
| Pacemakerleads, n (%) | 1931 (70.0) | 119 (70.0) | 35 (77.8) | ns |
| ICD-leads, n (%) | 654 (23.8) | 41 (24.1) | 8 (17.8) | ns |
| Left ventricle/coronary sinus leads, n (%) | 123 (4.5) | 7 (4.1) | 2 (4.4) | ns |
| VDD-leads, n (%) | 31 (1.1) | 1 (0.6) | 0 (0.0) | ns |
| SVC/Arrays, n (%) | 10 (0.4) | 2 (1.2) | 0 (0.0) | ns |
| **Total number of leads to be removed, n = 2964** | **2749** | **170** | **45** |  |
| Leads with active fixation, screw, n (%) | 1805 (65.7) | 93 (54.7) | 15 (33.3) | <0.0001 |
| Leads with active fixation, tines, n (%) | 757 (27.5) | 64 (37.6) | 28 (62.2) | <0.0001 |
| Passive fixation, unknown or other, n (%) | 187 (6.7) | 13 (7.6) | 2 (4.4) | ns |
| **Specific lead types** |  |  |  |  |
| Guidant 44-series, n (%) | 310 (11.3) | 18 (10.6) | 7 (15.6) | ns |
| Medtronic Starfix, n (%) | 5 (0.2) | 1 (0.6) | 0 (0.0) | ns |
| Medtronic Sprint Fidelis, n (%) | 183 (6.6) | 10 (5.9) | 2 (4.4) | ns |
| St.Jude Riata, n (%) | 24 (0.9) | 2 (1.2) | 0 (0.0) | ns |
| St.Jude Quickflex, n (%) | 15 (0.5) | 0 (0.0) | 1 (2.2) | ns |
| Medtronic Select Secure, n (%) | 14 (0.5) | 1 (0.6) | 0 (0.0 | ns |
| None of the specific types above, n (%) | 2198 (80.0) | 138 (81.1) | 35 (77.8) | ns |
| (%) = % of leads within each category of outcome, ns = not significant | | | | |

| **Table S10 Patient and procedure characteristics of possible and significant predictors of major complications** | | | | |
| --- | --- | --- | --- | --- |
|  | **Univariate analysis** | | **Multivariate analysis** | |
|  | **OR (95% CI)** | ***p*** | **OR (95% CI)** | ***p*** |
| Age, per 1 SD | 0.88 (0.60 – 1.33) | ns |  |  |
| Female gender | 2.73 (1.19 – 6.43) | 0.0177 | 3.03 (1.30 – 7.28) | 0.0103 |
| CIED history, two or more replacements/revisions | 4.28 (1.86 – 9.93) | 0.0008 |  |  |
| Infection yes/no | 1.48 (0.64 – 3.42) | ns |  |  |
| Left ventricular ejection fraction below 40%† | 0.54 (0.16 – 1.45) | ns |  |  |
| Dwelling time oldest lead per 5 years | 1.67 (1.27 – 2.14) | 0.0005 | 1.44 (1.07 – 1.90) | 0.0174 |
| Log number of leads to be removed | 2.31 (0.85 – 6.49) | ns |  |  |
| ICD-lead yes/no | 0.69 (0.27 – 1.63) | ns |  |  |
| One or more tined lead(s) | 2.65 (1.16 – 6.39) | 0.0214 |  |  |
| Fishing, snare yes/no | 4.82 (1.11 – 14.6) | 0.0377 |  |  |
| Single sheath yes/no‡ | 6.19 (0.83 – 46.05) | ns |  |  |
| Mechanical rotational sheath yes/no | 1.06 (0.31 – 2.86) | ns |  |  |
| Steel sheath yes/no | 5.30 (0.83 – 19.2) | ns |  |  |
| Lock wire‡ | 6.04 (0.81 – 44.98) | ns |  |  |
| Procedure time, per 15 minutes increase§ | 1.15 (1.07 – 1.23) | 0.0005 | 1.12 (1.03 – 1.21 ) | 0.0087 |
| Log X-ray time¶ | 1.38 (0.92 - 2.09) | ns |  |  |
| ns = not significant. †Registered for only 1557/1780 procedures (87.5%), 19/23 (82.6%) with major complication ‡Only one procedure in major complication group not involving use of single sheath and one without lock wire. Fischer`s exact test applied for OR. § registered for 1776 procedures, but for all 23 with major complications ¶1742 procedures, and 22/23 (95.6%) with major complications, ns = not significant | | | |  |
